# Cannabinomics in the flower of *Cannabis sativa*: a systematic review of extraction, analytical identification, and micro/nanoencapsulation methods for bioactive metabolites

**DOI:** 10.1186/s42238-025-00350-3

**Published:** 2025-11-25

**Authors:** Mateo Acosta Castaño, Juan Pablo Betancourt Arango, Francisco Javier Castellanos Galeano, Gonzalo Taborda Ocampo

**Affiliations:** 1 Center for Research, Innovation, Development and Technology Transfer, Department of Engineering, Universidad de Caldas, Manizales, Colombia; 2 Research Group in Chromatography and Related Techniques, Department of Chemistry, Universidad de Caldas, Manizales, Colombia

**Keywords:** Cannabinoids, Extraction, Food industry, Optimization, Pharmaceutical industry

## Abstract

**Introduction:**

The use of *Cannabis sativa* has evolved from textile applications in ancient times to a growing interest in its therapeutic and nutraceutical properties. Its regulation varies worldwide, with restrictions on ∆^9^-THC concentrations depending on the country. Cultivation factors, such as temperature, humidity and photoperiod, affect the concentration of their bioactive metabolites, among which phytocannabinoids have demonstrated impact on the biological regulation of the human organism. Their application in the pharmaceutical, cosmetic and food industries has prompted research into the optimization of their production and extraction.

**Objective:**

The purpose of this systematic review is to identify methodologies for the extraction, analysis and application of cannabinoids in various industries, focusing on agro-industrial transformation to increase their added value and optimize their therapeutic use.

**Methodology:**

A systematic search was performed in the Scopus database on November 14, 2024, identifying keywords and their synonyms for each research question, using Boolean operators. Studies published between 2015 and 2025 related to cannabinoid extraction, identification and application methodologies were included, excluding non-scientific papers. The PRISMA methodology was applied to filter and select articles.

**Results:**

The studies analyzed show that extraction and metabolomic analysis methodologies have gained relevance in recent years, especially for obtaining bioproducts for therapeutic purposes. It was identified that cannabinoids, mainly THC and CBD, have potential in the treatment of inflammatory, neurological and chronic pain diseases. In addition, the application of emerging technologies for the micro and nanoencapsulation of cannabinoids, optimizing their bioavailability, was evidenced. However, there are still gaps in the literature on the correlation between extraction operating conditions and the efficiency of the final product, which hinders its industrial scalability.

**Conclusions:**

The growing interest in *Cannabis sativa* research has led to the exploration of various techniques for the extraction and analysis of its metabolites. However, despite advances in laboratory methodologies, the industrial application of these processes remains a challenge. The lack of studies correlating operational variables with extraction efficiency limits the standardization of bioproducts. Future research should focus on articulating technology and applied science to establish production models to improve the traceability and safety of *Cannabis sativa* extracts, favoring their integration into the pharmaceutical and agro-industrial industry.

## Introduction

The term “Cannabis” is used worldwide to refer to the species *Cannabis sativa*,* Cannabis indica*, and *Cannabis rudelaris* (OMS 2018). Historically, the most widely cultivated variety of cannabis has been hemp, with records of its use dating back to 10,000 BC in Central Asia (Yazici 2023), primarily for the production of plant fiber. However, in the last five years, compositional analysis and the investigation of the plant’s therapeutic potential have gained increasing relevance in the pharmaceutical, cosmetic, and food industries (Capriotti et al. 2021). Emperor Sheng Nung, considered the father of Chinese medicine, described marijuana as a plant of great importance for the treatment of bodily ailments—a view supported by ancient documents such as the Ebers Papyrus, which mentions its medicinal use in ancient Egypt (Covarrubias-Torres 2017).

According to the Food and Agriculture Organization of the United Nations (FAO), global production of *Cannabis sativa* increased by 28% between 2016 and 2020, with an average of 4,325.2 tons of dried flowers produced annually. The main producers include the United States, Canada, Germany, Italy, and Uruguay (Piñeros 2022). In Colombia, cannabis production is regulated by national legislation that governs its cultivation, processing, and commercialization (Table 1). In this context, the agro-industrial transformation of cannabis is oriented toward the development of strategies to increase its added value and optimize its therapeutic use—especially through the formulation of pharmaceutical and nutraceutical bioproducts.

**Table 1:** Regulatory acts in some countries of the Americas for the cultivation and disposal of cannabis derivatives

Country or Region	Legal Framework and Regulations	Maximum Δ^9^-THC* Allowed	Permitted Use	Reference	
United States	2018 Farm Bill	≤ 0.3% (industrial hemp)	Industrial hemp permitted; medicinal and recreational cannabis legality varies by state	(Ministerio de Salud y Protección Social de Colombia 2022) (Falkner et al. 2023)	
Canada	Cannabis Act (2018)	Up to 30 g/day recreationally	Recreational and medicinal cannabis legal	(Ministerio de Salud y Protección Social de Colombia 2022) (Crépault 2018)	
Uruguay	Law 19.172 (2013); subsequent regulations	15–20% THC allowed in products	Recreational and medicinal use legal	(Zuleta, et al. 2021) (Alvarez, et al. 2023)	
Mexico	Supreme Court ruling (2021); COFEPRIS manages permits	Up to 1% authorized	Recreational and medicinal use legal	(Aguinaco, and Barra 2017)	
Colombia/Peru/Ecuador	Decree 811/2021 – Resolution 227/2022	≤ 1% THC (non-psychoactive)	Only non-psychoactive hemp permitted	(Staff 2022)	
Argentina	Incorporates provisions from U.S. Farm Bill	≤ 0.3% for hemp	Industrial hemp authorized; restricted medicinal use	(Romero 2019)	
Brazil	Medical cultivation authorized since 2024; hemp regulation pending	Medicinal use < 0.2% THC	Medicinal use permitted; recreational use prohibited		(Martins, and Posso 2023)
Thailand	Regulations allow hemp (≤ 0.2% THC), medicinal use permitted; recreational use illegal	≤ 0.2% THC	Hemp and industrial uses; controlled medicinal use		(Tanguay 2024)
Spain	EU Regulation No. 1307/2013; cultivation allowed under strict conditions	≤ 0.3% THC	Cultivation of hemp allowed; medicinal cannabis permitted via magistral formula only; recreational use illegal		(Arana 2019)
Portugal	Law No. 33/2018; Decree 8/2019	≤ 0.2% THC (for hemp)	Medicinal cannabis legal; cultivation requires INFARMED authorization		(Farber, et al. 2024)
France	EU Regulation + French Public Health Code; liberalized in 2022	≤ 0.3% THC	Hemp permitted; medicinal cannabis in pilot program; recreational use illegal		(Wallez, et al. 2024)

*Δ^9^-THC = Δ^9^-tetrahydrocannabinol

Cultivation characteristics significantly impact the chemical composition of cannabis, particularly the concentration of secondary metabolites (terpenes, cannabinoids and flavonoids in particular) (Capriotti et al. 2021; Yazici 2023). Factors such as planting method, soil type, and environmental conditions (temperatures between 20 °C and 25 °C, relative humidity between 60% and 70%, and a photoperiod brightness range of (400–700 nm) (Ángeles-López et al. 2014), are determinants in the production of key chemical families, including terpenophenols (cannabinoids), terpenes, flavonoids, alkaloids, stilbenes, amides, and lignanamides (Ángeles-López et al. 2014; Kornpointner et al. 2021).

Cannabinoids—the main bioactive metabolites of *Cannabis sativa*—interact with the human endocannabinoid system (ECS), identified in the early 1990s. This system regulates essential physiological functions such as cognition, memory, pain perception, sleep, appetite, gastrointestinal processes, and immune response (Wayne 2018). From a chemical standpoint, cannabinoids such as Δ^9^-tetrahydrocannabinol (THC) and cannabidiol (CBD) possess phenolic and alkyl side chain functional groups that allow them to bind to cannabinoid receptors CB1 and CB2. THC has high affinity for CB1 receptors, predominantly located in the central nervous system, and is responsible for the plant’s psychoactive effects. In contrast, CBD shows lower affinity for both receptors but acts as an indirect modulator, exerting therapeutic effects without psychotropic activity (Calapai et al. 2022).

For example, Huang et al. (2023) highlight the use of metabolites derived from hemp root extracts—including cannabinoids, phytosterols, triterpenoids, and unsaturated chain hydrocarbons—for the treatment of inflammatory conditions. Furthermore, it has been reported that cannabis contains over 60 phytocannabinoids, with THC and CBD being the most abundant across species. These compounds have shown efficacy in reducing anxiety, managing nausea and dizziness, increasing appetite, and contributing to the treatment of various mental health conditions (Gallego-Álvarez 2017; Huang et al. 2023).

Bourke et al. (2022) emphasize that cannabinoids are distributed across various anatomical parts of the cannabis plant in variable concentrations. They possess therapeutic potential as analgesics, antiepileptics, muscle relaxants, anxiolytics, neuroprotectants, antipsychotics, anti-inflammatories, antioxidants, and agents in palliative cancer care (Bourke et al. 2022). Each Cannabis species exhibits structural and biochemical specificity across roots, stems, leaves, and flowers, making possible the production of both extracts and pulverized materials. These characteristics define the industrial uses of each plant part, particularly in the pharmaceutical, cosmetic, and food sectors.

Cannabis has been used for thousands of years in therapeutic, recreational, and cultural contexts (Hanuš et al. 2016; Hesami et al. 2020). Medicinal preparations derived from cannabis are rooted in Ayurvedic medicine, where three main forms are described: “bhang” (prepared from dried leaves), “ganja” (from dried female flowers), and “charas” (resin extracted from the plant’s leaves) (Lawrence and Lawrence 2022). These traditional forms are not limited to the pharmaceutical sector but also intersect with the food, cosmetics, and recreational industries (Ángeles-López et al. 2014). Being a relatively young and emerging industry with long-term global potential, current agro-industrial transformation studies focus on harnessing cannabis-derived metabolites for the treatment of chronic and neuropathic pain, including fibromyalgia (Pusiak et al. 2021).

According to Canavan et al. (2022), cannabis varieties with high fiber content—used for the production of construction materials—offer higher biomass yields and contribute to environmental remediation. Conversely, varieties cultivated for pharmacological or psychoactive use typically yield less biomass but exhibit higher concentrations of bioactive metabolites (Hesami et al. 2020; Acosta and Almirall 2021; Canavan et al. 2022). Analytically, the different parts of the cannabis plant demonstrate a wide range of applications, classified as seeds, leaves, and flowers. In the case of seeds, they are mainly used in the production of oils for varnishes and lubricants, as well as in the generation of biofuels and biomass (Myles et al. 2016). The leaves, on the other hand, are used to extract fibers used in the production of paper, textiles, bioplastics, and construction materials, demonstrating their potential as a sustainable resource for various industrial markets (Dalle Vacche et al. 2021; Chatzimitakos et al. 2024).

The flowers constitute the component with the greatest diversity of applications, being used in the production of extracts for medicinal purposes and in maceration for the manufacture of cigarettes (Acosta & Admiral, 2021). Likewise, solutions with relevant pharmacological properties are obtained, including immunosuppressive, anti-inflammatory, antidepressant, bronchodilator, analgesic, and antineoplastic effects, among others, with a wide variety of uses (Chatzimitakos et al. 2024; Dalle Vacche et al. 2021). These have led to the development of ointments intended for palliative care, positioning the plant as a highly valuable resource in both the pharmaceutical industry and the healthcare field.

Avello et al. (2017) describe cannabinoids as complementary treatments with neuroprotective effects and potential in managing tumor pathologies, anxiety, and depression. Some companies specialize solely in cultivation, while others integrate processing activities to extract compounds of interest. To obtain high-value extracts, various extraction methodologies are employed, taking into account operating conditions, investment cost, extraction efficiency, and production yield. These factors are critical for selecting the most suitable technologies for the production of pharmaceutical, food, and cosmetic inputs (Avello et al. 2017), among other bioproducts (Christodoulou et al. 2022). In light of this background, the present systematic review addresses the following research questions: What are the most explored methodologies and operational conditions identified for the extraction of cannabinoids from *Cannabis sativa* flowers? What methodologies and protocols are used for the analytical identification of cannabinoids contained in the flowers of *Cannabis sativa*? What are the reported applications in the pharmaceutical and food (nutraceutical) industries for extracts obtained from *Cannabis sativa* flowers?

## Methodology: search for information

**Fig. 1:**
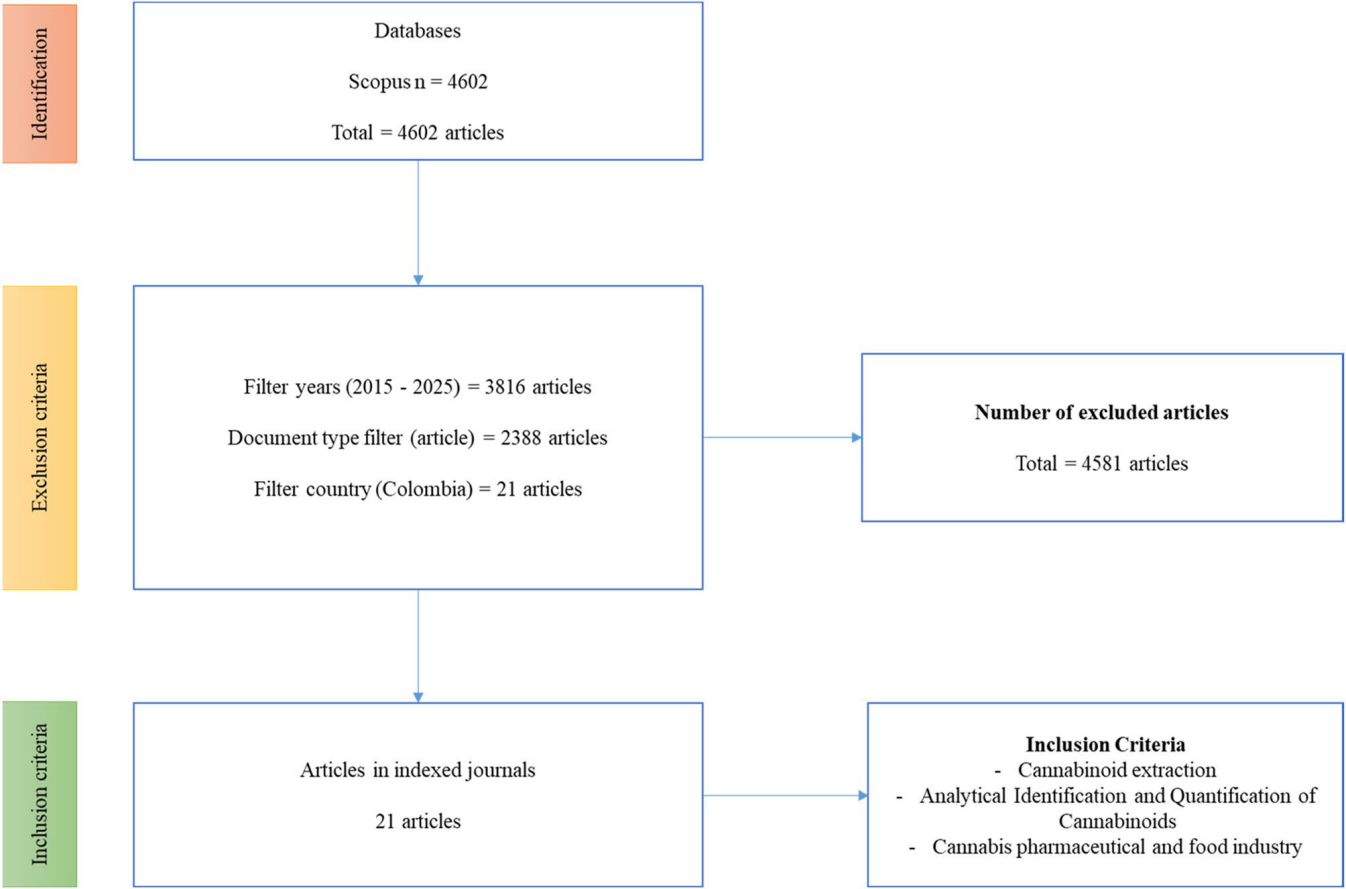
Diagram of PRISMA methodology used to search for information

The information search for this systematic review was conducted on November 14, 2024, using the Scopus database. The strategy followed the methodology proposed by Moreno et al. (2018), which allowed us to extract and refine keywords for each research question, including their relevant synonyms, in order to maximize the retrieval of pertinent literature. The Boolean operators “AND” (to link categories) and “OR” (to include synonyms) were used to construct the search string (Moreno et al. 2018). The search strategy was delimited by applying specific exclusion criteria, including: Time period: 2015 to 2025, Language: English, Document type: Peer-reviewed scientific articles, Geographic filter: Studies with institutional affiliation in Colombia (to narrow focus and identify national research contributions). Inclusion criteria focused on studies that addressed at least one of the following: Methodologies and operational protocols for the extraction of cannabinoids from *Cannabis sativa*, Analytical techniques and omics-based tools for the identification and characterization of phytocannabinoids. Applications of these compounds in the pharmaceutical, nutraceutical, or food industries. To ensure methodological transparency and minimize bias in the selection of studies, the PRISMA protocol was applied (Fig. 1) (Page et al. 2021), including a multi-phase screening process (identification, screening, eligibility, and inclusion). For the development of this research, the following search equation was used:

((Methodology OR protocol OR method OR techniques OR metabolomic OR “biochemical pathway”) AND (extraction OR separate OR divide OR synthesis OR “metabolite production” OR encapsulation OR microencapsulation OR nanoencapsulation OR qualification OR identification OR quantification OR concentration OR measurement) AND (terpenophenol OR cannabinoid OR terpene OR isoprenoid OR metabolite) AND (Cannabis OR marihuana OR marijuana OR hemp) AND (“pharmaceutical uses” OR “food uses” OR “uses of cannabis” OR “cannabis extract” OR “cannabinoids extract”))

In addition to PRISMA, we conducted an automated risk of bias assessment using the ROBIS (Risk of Bias in Systematic Reviews) tool, adapted for large-scale screening. This evaluation was implemented in RStudio using keyword- and metadata-based heuristics to classify studies according to their methodological rigor. ROBIS assessed four domains: Eligibility criteria clarity, Study identification and reporting, Data collection and appraisal, Synthesis of findings (Lunny et al. 2025).

**Fig. 2:**
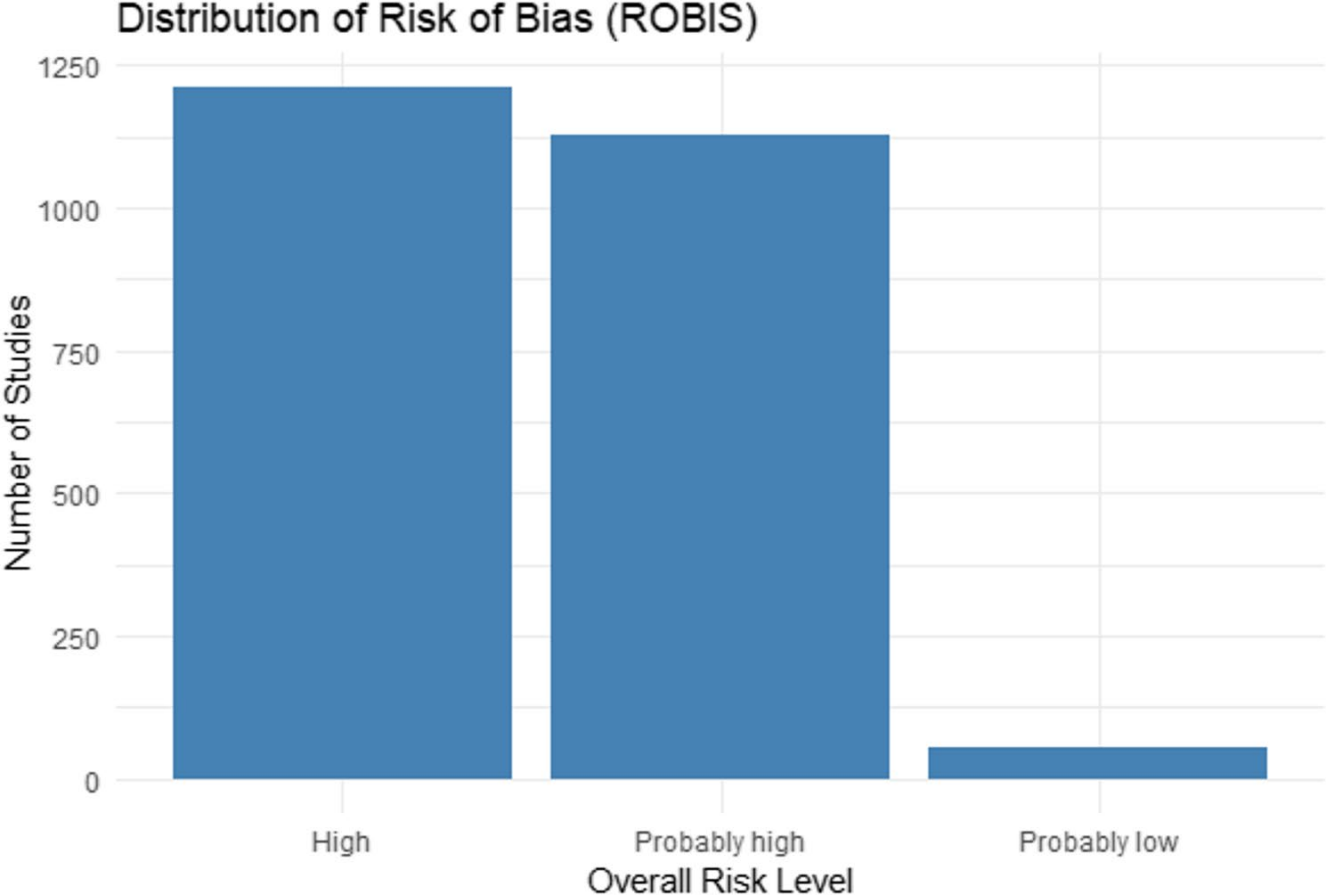
Distribution of risk of bias among studies based on the ROBIS tool

Each study received an overall risk rating: high, probably high, or probably low. As shown in Fig. 2, most of the excluded studies were classified as high or probably high risk, due to limitations such as lack of protocol statement, unclear inclusion criteria, or insufficient methodological details. Only a small proportion met the minimum methodological transparency required for a probably low risk classification. We identified 54 articles with a probably low bias, of which 21 articles were selected that met the inclusion criteria, responding on key aspects such as extraction, identification, quantification of cannabinoids, as well as their respective pharmaceutical applications in the food industry.

## Chemical extraction of terpenophenols from *Cannabis sativa*

Pattnaik et al. (2022) point out that cannabis concentrates are products with high concentrations of cannabinoids and terpenes. There are different ways to obtain them, but all of them are based on the separation of the trichomes present in the *Cannabis sativa* flowers (Champagne and Boutry 2016; Pattnaik et al. 2022). These trichomes are small, sticky crystals that coat the buds and contain all the chemicals that give cannabis its distinguishing properties (Richins et al. 2018). The main difference between concentrate and extract is that while a concentrate can be produced from a wide variety of trichomes and without the need to use a solvent, extracts are made from a single concentrate part with the help of a solvent such as alcohol, carbon dioxide, among others.

The extraction techniques present diverse physicochemical and particular characteristics depending on the methodologies employed and the purpose for which the extract is intended, as expressed in the specific industrial sector to which the product is directed (López-Olmos et al. 2022). Therefore, the operating conditions under which the processes are carried out are essential to achieve effective extraction. Factors such as temperature, system pressure, extraction time, solvent polarity and type, agitation, and particle size are determinants in the physicochemical-bromatological characterization of the plant material taken as raw input (Song et al. 2023). These parameters directly influence the yield, selectivity, purity, and stability of the bioactive compounds extracted.

Over the last decade, several extraction methodologies have been reported in the scientific literature for the recovery of bioactive compounds from *Cannabis sativa*, particularly from flowers. These techniques differ in their level of efficiency, selectivity, economic feasibility, and scalability, and have proven fundamental for the production of value-added products in various sectors. Among the most widely used methods are solvent extraction (ethanol, methanol, butane, propane), supercritical fluid extraction (SFE) (Lazarjani et al. 2021), ultrasound-assisted extraction (UAE), microwave-assisted extraction (MAE) (Radoiu et al. 2020), pressurized liquid extraction (PLE) (Serna-loaiza et al. 2020), and traditional techniques such as steam distillation and hydrodistillation (Sainz Martinez, et al. 2023).

The selection of the extraction method is conditioned by the target cannabinoid, its thermal sensitivity, solubility, and intended application (Al Ubeed et al. 2022). For instance, some methods preserve acidic precursors such as THCA and CBDA, while others induce decarboxylation, resulting in active forms such as THC and CBD. The reproducibility of results across studies is often limited by variations in chemotype, cultivar, drying methods, and sample pre-treatment, making standardization a critical challenge in cannabis extraction research.

Table 2 presents a comparison of extraction techniques frequently reported in the literature, including operational parameters (solvent, temperature, time, pressure), and percentage yield. This table aims to contextualize methodological variability and facilitate critical comparison between technologies for researchers and industrial stakeholders (Al Ubeed et al. 2022). However, it should be noted that differences in cannabis chemotypes and biomass characteristics, as well as variations in extraction scale and instrumentation, may affect the direct comparability of the results. Therefore, these values should be interpreted with caution and within the context of each individual study’s design.

**Table 2:** Methodological developments and operational variables for different extraction processes in the development of time

Methodology	Operating conditions	Performance	Reference
Solvent extraction	Temperature: 110 °C Water Time 3 h	0.015% p/p total cannabinoids	(Naz et al. 2017)
	Temperature: 120 °C Water Time 3 h	0.020% p/p total cannabinoids	
	Temperature: 130 °C Water Time 3 h	0.025% p/p total cannabinoids	
Solvent extraction	Temperature: 110 °C Water Time 3 h	0.035% p/p total cannabinoids	(Citti et al. 2016)
	Temperature: 120 °C Water Time 3 h	0.030% p/p total cannabinoids	
	Temperature: 130 °C Water Time 3 h	0.026% p/p total cannabinoids	
Solvent extraction by means of maceration	Temperature: (20–60) °C RPM: 600 Solvent: 2-hydroxypropylcyclodextrin	0.12% p/p CBD 1.55% p/p THC	(Casiraghi et al. 2018)
	Temperature: (40–80) °C Solvent: Absolute ethanol Time: 30 to 90 min	8.17% CBD 28.14% THC	(Monton et al. 2019)
Supercritical fluid extraction	Temperature: 40 °C Pressure: 1160.3 psia Solvent: Carbon dioxide	0.031% p/p CBDA	(Naz et al. 2017)
	Temperature: 54 °C Pressure: 2465.64 psia Solvent: Carbon dioxide Co-solvent: Ethanol	2.92 ± 1.05 CBDA	(Rovetto and Aieta 2017)
	Temperature: 40 °C Pressure: 2175.57 psia Solvent: Carbon dioxide Co-solvent: Ethanol	26.36% p/p CBDA	(Gallo-Molina et al. 2019)

## Analytical identification of cannabinoids in *Cannabis sativa*

The identification and characterization of cannabinoids present in *Cannabis sativa* has become a crucial aspect for scientific research, quality control, and regulatory compliance in products derived from this plant. Currently, several instrumental techniques have been employed for the determination of these compounds, encompassing chromatographic, spectroscopic, and combined high-resolution analytical approaches.

Chromatographic techniques coupled with mass spectrometry have proven to be fundamental tools in the identification of cannabinoids and other metabolites in cannabis samples. Among the most widely used methods are gas chromatography (GC) and high-performance liquid chromatography (HPLC), often combined with various detectors. Specifically, GC has been extensively used in conjunction with detectors such as flame ionization (GC-FID), conventional mass spectrometry (GC-MS) (Nahar et al. 2023), and tandem mass spectrometry (GC-MS/MS), as well as more advanced platforms like two-dimensional gas chromatography coupled to time-of-flight mass spectrometry (GCxGC-TOF-MS) (Pourseyed Lazarjani et al. 2020). These techniques offer high sensitivity, selectivity, and the ability to resolve complex cannabinoid profiles, although GC requires derivatization of acidic cannabinoids to avoid decarboxylation during analysis (Ciolino et al. 2018; Anthony-Macherone, 2020).

On the other hand, HPLC allows for the identification and quantification of cannabinoids without prior derivatization, preserving thermolabile compounds such as THCA and CBDA. HPLC can be coupled with ultraviolet detection (HPLC-UV) (Zivovinovic et al. 2018), diode array detectors (HPLC-DAD**)**, or various mass spectrometry configurations including electrospray ionization (HPLC-ESI-MS/MS) and quadrupole time-of-flight (HPLC-QTOF-MS). Supercritical fluid chromatography (SFC) has also gained importance due to its efficiency, selectivity, and reduced solvent consumption (Pilařová et al. 2022). Methods such as SFC-MS and SFC-HF-QTOF-MS/MS have enabled precise and fast identification of cannabinoids, making them suitable for high-throughput and quality assurance workflows (Kołodziej et al. 2024).

Non-chromatographic analytical techniques have also been employed for the detection and structural elucidation of cannabinoids and other secondary metabolites in *Cannabis sativa*. Fourier transform infrared spectroscopy (FT-IR) and Raman spectroscopy have been used for rapid and non-destructive screening (Tay et al. 2022; Duchateau et al. 2025). Meanwhile, high-resolution mass spectrometry techniques such as matrix-assisted laser desorption/ionization time-of-flight (MALDI-TOF-MS) and orbitrap-MS have shown strong performance in profiling complex mixtures, particularly when integrated with chemometric tools (dos Santos et al. 2019). Nuclear magnetic resonance (NMR) has also played a key role in cannabinoid analysis (Barthlott et al. 2021). Techniques such as ^1^H-NMR, ^13^C-NMR, two-dimensional DOSY-NMR, and liquid chromatography-NMR (LC-NMR) have been applied for the elucidation of cannabinoid structures, even in unfractionated samples (Colella et al. 2022). Additionally, capillary electrophoresis coupled to mass spectrometry (CE-MS) provides an alternative with high separation efficiency and low sample consumption (Blebea et al. 2023).

Validation and standardization remain challenges across these analytical platforms. Differences in ionization techniques, detection thresholds, and sample preparation protocols can lead to variability in results between laboratories. The lack of certified reference materials for minor cannabinoids also limits reproducibility in omics-scale studies. However, efforts to harmonize methodologies and implement robust quality control systems are underway, particularly in pharmacopoeia-driven research. For instance, the characterization of aroma compounds in dried cannabis flowers has been carried out using solid phase microextraction with headspace coupled to gas chromatography–mass spectrometry (HS-SPME-GC-MS), enabling the identification of 75 volatile compounds. These include alcohols, aldehydes, esters, ketones, monoterpenes, monoterpenoids, sesquiterpenes, and sesquiterpenoids (Cajigas et al. 2024). Through multivariate statistical analyses, such as hierarchical cluster analysis (HCA) and principal component analysis (PCA) (Gambardella et al. 2021), 19 clusters were grouped into five volatile chemotypes. Moreover, partial least squares discriminant analysis (PLS-DA) allowed the identification of 20 discriminant markers, providing tools for differentiation and quality control based on secondary metabolite profiles (Janta and Vimolmangkang 2024). A summary of the main phytocannabinoids reported in *Cannabis sativa* is presented in Table 3, including major cannabinoids, their acidic precursors, degradation products, and minor cannabinoids with emerging pharmacological interest.

**Table 3:** Main cannabinoids reported in *Cannabis sativa*

Composite number	Main phytocannabinoids	Cannabinoid acids (precursor forms)	Degradation products	Other minor cannabinoids
1	Δ^9^- Tetrahydrocannabinol (Δ^9^-THC)	Tetrahydrocannabinolic acid (THCA)	Cannabinol (CBN)	Tetrahydrocannabiforol (THCP)
2	Δ^8^- Tetrahydrocannabinol (Δ^8^-THC)	Tetrahydrocannabivaric acid (THCVA)	Cannabielsoin (CBE)	Cannabidiforol (CBDP)
3	Tetrahydrocannabivarin (THCV)	Cannabidiolic acid (CBDA)	Cannabicyclol (CBL)	Cannabichromevarina (CBCV)
4	Cannabidiol (CBD)	Cannabidivarinic acid (CBDVA)	Cannabifurans (CBF)	Cannabigerovarina (CBGV)
5	Cannabidivarin (CBDV)	Cannabigerolic acid (CBGA)	Cannabiripsol (CBR)	Cannabicitran (CBT)
6	Cannabinol (CBN)	Cannabichromenolic acid (CBCA)		
7	Cannabigerol (CBG)			
8	Cannabichromene (CBC)			

On the other hand, the analysis of hemp (*Cannabis sativa*) by gas chromatography coupled with mass spectrometry (GC-MS) has proven to be an effective analytical tool for the separation and identification of cannabinoids, allowing the characterization of ten compounds through comparison with standards and their respective quantification (Prato et al. 2022). In addition, several studies have evaluated diverse methodologies for the detection of cannabinoids in biological and environmental matrices. For example, Shah et al. (2019a, b) conducted a review of techniques used for detecting cannabinoids in hair samples, highlighting the application of several instrumental methods, including GC-MS (De Prato et al. 2022), electron impact ionization mass spectrometry (GC-EI/MS), tandem mass spectrometry (GC-MS/MS), gas chromatography with negative chemical ionization (GC-MS-NCI), tandem GC-MS with negative chemical ionization (GC-MS/MS-NCI) (Moore et al. 2001), positive chemical ionization (GC-MS/MS-PCI), triple quadrupole GC with NCI (GC-NCI-(QqQ)-MS/MS), and two-dimensional gas chromatography (2D-GC) (Shah et al. 2019).

Similarly, the use of liquid chromatography combined with high-resolution mass spectrometry (LC-MS, LC-MS/MS, LC-ESI-QTOF-MS), ultra-high pressure liquid chromatography (UHPLC), and supercritical fluid chromatography (UHPSFC) has been reported in various matrices. Emerging techniques such as desorption atmospheric pressure resonance ionization mass spectrometry (DART-MS), multiple reaction monitoring (MRM) chromatography (Shah et al. 2019), and MALDI-MS have also been incorporated into cannabinoid detection workflows. Therefore, advancements in cannabinoid identification methodologies continue to evolve through the integration of metabolomic, volatilomic, and transcriptomic approaches (Ma et al. 2024), contributing to the development of more robust differentiation strategies and quality control systems. These multi-platform strategies are essential for the comprehensive identification and confirmation of bioactive metabolites in cannabis, especially in the context of increasingly stringent industrial and regulatory standards.

## Omics approaches in cannabis research

More than 500 compounds have been identified in Cannabis, including over 100 cannabinoid species with both psychotropic and pharmacological effects (Schaiquevich et al. 2020). Among these, cannabidiol (CBD) and Δ^9^-tetrahydrocannabinol (THC) are the most extensively studied and represent the metabolites found in highest concentrations in pharmaceutical-grade cannabis varieties. Additionally, more than 120 types of terpenes—responsible for aroma and also acting as cannabinoid potentiators—have been characterized, along with flavonoids, which modulate the biological activity of cannabinoids, and other chemical families such as alkaloids, stilbenoids, and lignanamides (Ángeles-López et al. 2014).

**Fig. 3:**
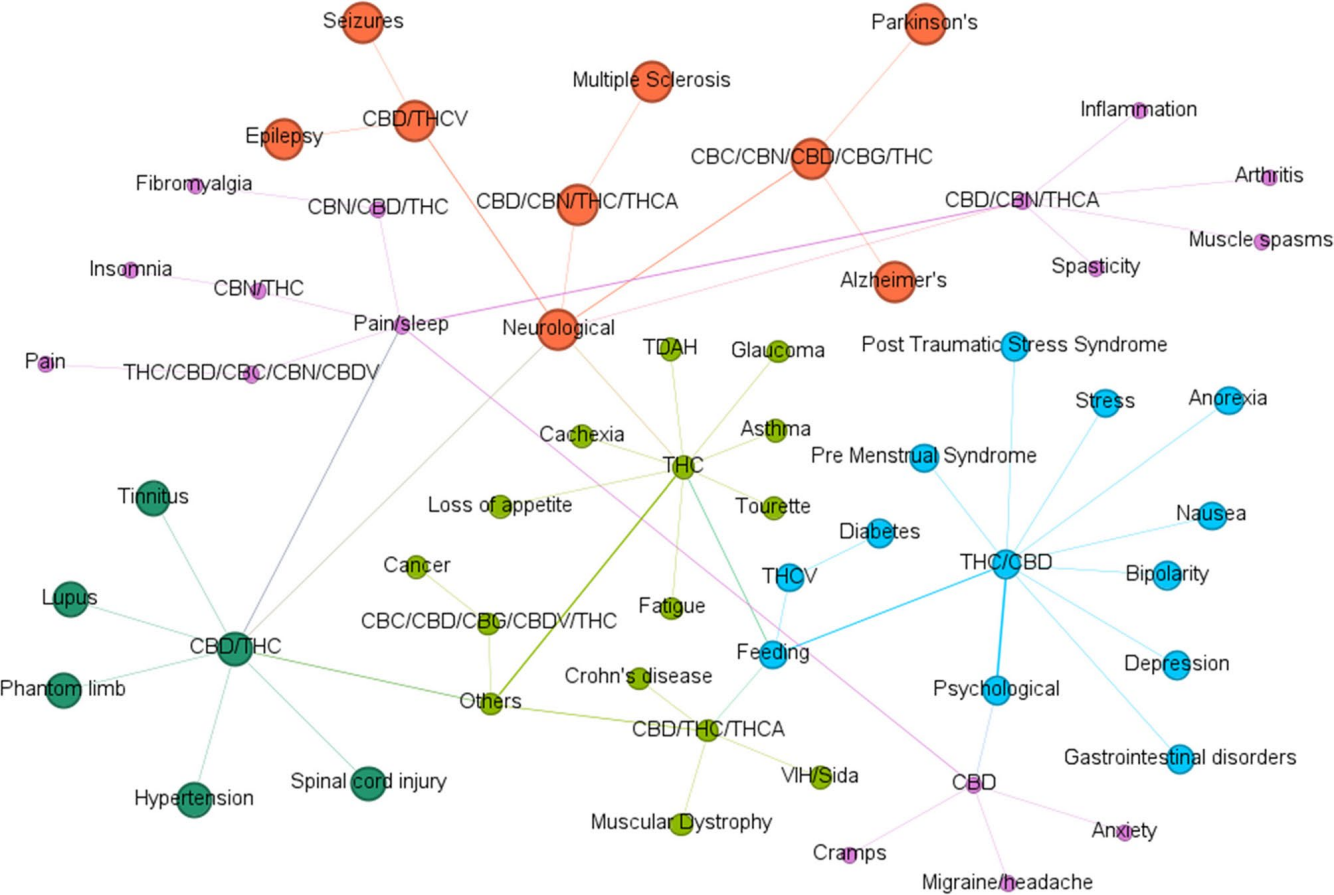
Compositional relationship between cannabinoids and diseases in humans

Figure 3 illustrates the relationship between major cannabinoids and their physiological impacts, especially in the context of therapeutic applications. Of particular interest is the widespread influence of CBD on multiple health conditions, which supports its growing role as a focal point for pharmaceutical research due to its high concentration in dried flower biomass of medical cultivars (Ahmed et al. 2021; Casati et al. 2022). Moreover, Fig. 3 highlights the range of physiological systems modulated by cannabinoids, including the gastrointestinal, genitourinary, nervous, and cardiovascular systems, as well as structures like bones, skin, and eyes—making cannabis derivatives increasingly relevant in the cosmetic industry (Muñoz-Ramírez 2021).

The endocannabinoid system (ECS) in the human body functions in parallel with the opioid system (responsive to morphine and codeine) and includes cannabinoid-specific receptors (CB1 and CB2) that regulate a wide array of physiological processes (Console-Bram et al. 2017; Muñoz-Ramírez 2021). Phytocannabinoids derived from plants interact with these receptors and modulate the body’s natural production of endocannabinoids, thereby playing a key role in homeostatic balance (Barragán et al. 2020). The ECS is fundamental to neural function and is involved in regulating pain, appetite, digestion, sleep, mood, inflammation, and memory.

Basto et al. (2021), emphasized that endocannabinoids are naturally produced by the human body, acting on the same receptors targeted by phytocannabinoids such as THC and CBD (Basto et al. 2021; Duncan et al. 2024). While many minor cannabinoids exist, they remain underexplored. In this context, Ahmed et al. (2021) proposed a GC-MS method for the detection of cannabinoids like cannabigerol (CBG), cannabichromene (CBC), and cannabinol (CBN) (Ahmed et al. 2021). Despite their typically low concentrations, these compounds contribute to the overall phytotherapeutic activity of cannabis extracts and have been included in emerging bioproduct formulations (Gibson et al. 2024). The presence of CB1 receptors in the brain explains the modulation of physical and behavioral functions, such as motor control, heart rate, appetite, emotional processing, nausea, memory, and executive decision-making.

The metabolomic profile of *Cannabis spp.* is highly diverse, including numerous cannabinoid-like molecules with proven pharmacological activity, as well as anti-inflammatory, antimicrobial, and antibacterial effects against both Gram-positive and Gram-negative bacteria (Monyela et al. 2024). However, certain extracts may induce side effects like cognitive dysfunction and motor impairment. In response to this concern, a recent study applied a non-targeted metabolomic approach using LC-MS/MS and ¹H-NMR to identify metabolic changes in rats exposed to full-spectrum cannabis extracts. The results revealed increased serum levels of phenylalanine and long-chain acylcarnitines, and decreased levels of butyric acid and lysophosphatidylcholines, which may serve as predictive biomarkers for cannabis-induced behavioral effects (Maayah et al. 2022). In another study, the concentrations of tetrahydrocannabinolic acid (THCA) and cannabidiolic acid (CBDA) were monitored in samples collected at different harvest stages using non-targeted metabolomics based on HPLC and NMR, demonstrating variations in metabolite content even within the same *Cannabis sativa* cultivar (Castro et al. 2023).

Metabolomics has also been used to assess product authenticity and regulatory compliance. Due to the complexity of the cannabis matrix and the diversity of its metabolites, non-targeted metabolomic approaches are essential for tracking both key bioactive compounds and potential contaminants (Jadhav et al. 2023). From this effort arises the authentomics strategy, which involves comparing current analytical data with historical metabolomic datasets. This facilitates the evaluation of product quality and the verification of compliance with international quality standards, including those set by the International Organization for Standardization (ISO) (Jadhav et al. 2023).

## Volatilomics in *Cannabis sativa*

The omics sciences have been progressively expanding, diversifying, and adapting to various biological systems of different scales. This evolution has given rise to multiple branches and sub-disciplines that focus on specific molecular targets or biological contexts. Within the broader field of metabolomics, specialized subfields have emerged, such as peptidomics, lipidomics, and volatilomics (Betancourt-Arango et al. 2024). Volatilomics specifically refers to the comprehensive study of volatile organic compounds (VOCs)—small molecules of low molecular weight that are naturally emitted or externally induced from biological matrices. The analysis of VOCs is increasingly relevant across diverse disciplines, including medical diagnostics, food science, environmental monitoring, and, notably, cannabis research (Betancourt-Arango et al. 2025). As volatilomics evolves, additional sub-branches have emerged based on the biological matrix or application focus, including exhalomics, xenovolatilomics, and, in the case of *Cannabis sativa*, cannabinomics.

Volatilomic studies in cannabis involve identifying the optimal conditions for extracting the largest possible spectrum of secondary metabolites from the plant matrix, followed by the application of appropriate analytical techniques for their separation, detection, and identification (Cicaloni et al. 2022). These typically include solid-phase microextraction (SPME) coupled with gas chromatography–mass spectrometry (GC-MS), which allow high-resolution analysis of complex volatile profiles (Taiti et al. 2022).

Once the chemical constituents are identified and validated, researchers often apply chemometric techniques to interpret complex volatilomic data. These techniques include multivariate statistical analyses, both supervised (PLS-DA, LDA) and unsupervised (PCA, HCA), which are essential for sample classification, quality control, and varietal discrimination (Noshad et al. 2023). In recent years, machine learning models have shown increasing utility in volatilomics for tasks such as: Differentiating samples from the same species based on environmental or cultivation variables, classifying samples by geographical origin or genetic background, discriminating between contaminated and non-contaminated samples, and identifying authenticity markers for regulatory or forensic purposes (Betancourt-Arango et al. 2024). In the context of *Cannabis sativa*, volatilomic profiling plays a critical role in strain differentiation, standardization of aroma profiles, and the identification of quality markers linked to therapeutic or industrial potential. As such, volatilomics represents a valuable analytical layer in the multi-omics characterization of cannabis and its derivatives.

## Cannabinomics

The term cannabinomics has emerged in response to the growing specialization within omics sciences, referring specifically to the systematic and comprehensive analysis of the full cannabinoid profile and their derivatives in various biological matrices and cannabis-derived products (Santos et al. 2023). This concept is understood as a sub-discipline of metabolomics and volatilomics, focused on the identification, quantification, and characterization of cannabinoids and their metabolites through advanced analytical platforms such as gas chromatography-mass spectrometry (GC-MS) and liquid chromatography-mass spectrometry (LC-MS). Cannabinomics aims to go beyond the quantification of major cannabinoids (THC and CBD), expanding towards: Comprehensive chemical profiling of cannabis strains, investigation of cannabinoid metabolism, study of interactions with other bioactive compounds, detection of adulterants or contaminants, classification and authentication of extracts or finished products (Santos et al. 2023).

This discipline often integrates untargeted metabolomics workflows using mass spectrometry-based techniques combined with statistical or chemometric analyses to differentiate cultivars and identify biomarkers. For instance, through LC-MS and multivariate analysis, one study differentiated 20 cultivars of *Cannabis sativa* and classified them into chemotypes I, II, and III, according to their cannabinoid content (Vásquez-Ocmín et al. 2021). These findings underscore the utility of cannabinomics as a complementary tool to discover minor bioactive compounds and define differentiation markers. Cannabinomics also supports the taxonomy of cannabis into chemovars, enabling the discovery of new varieties and the identification of therapeutic principles for pharmaceutical, nutraceutical, and cosmetic uses, as well as the optimization of crop management for higher yields (Aliferis et al. 2020).

Although hemp (*Cannabis sativa* with low Δ^9^-THC levels) has been historically cultivated for fiber, its phytochemical profile remains underexplored. Given the increasing interest in hemp-derived pharmaceuticals and nutraceuticals, cannabinomics has become a crucial tool for characterizing compounds beyond THC and CBD. In a study using LC-HRMS, a total of 54 phytocannabinoids, 134 flavonoids, and 77 phenolic acids were identified across seven industrial hemp cultivars grown in different regions of Italy (Cerrato et al. 2023). These compounds played a key role in chemovar discrimination and were linked to specific biological activities. A low-level data fusion model demonstrated that cannabinomics could integrate minor or less-studied metabolites for sample classification and standardization.

From a biosynthetic perspective, phytocannabinoids are isoprenylated resorcinyl polyketides, produced mainly in the glandular trichomes of *Cannabis sativa*, and are responsible for modulating psychotropic and pharmacological responses via CB receptors in the central nervous system (Sommano et al. 2022). While aliphatic ketidic cannabinoids appear to be unique to cannabis, phenethyl-type phytocannabinoids have been found in other species of angiosperms, bryophytes, and fungi, expanding the scope of cannabinoid research beyond the genus Cannabis (Sommano et al. 2022).

## Micro/nanoencapsulation of cannabinoids

One of the main challenges in the micro- and nanoencapsulation of cannabinoids stems from their thermolabile and lipophilic nature, which makes them vulnerable to degradation by heat, light, oxidation, and gastrointestinal enzymes (Lindholst 2010; Fraguas Sánchez et al. 2020). These physicochemical limitations can reduce their bioactivity and hinder the development of stable formulations for oral or topical use. As a result, various encapsulation strategies have been developed to overcome issues related to compound degradation, uncontrolled release, dosage accuracy, masking of bitter or undesirable flavors, and the preservation of functional aroma and flavor compounds.

In this context, micro/nanoencapsulation offers multiple benefits: Enhanced chemical and thermal stability of cannabinoids, improved bioavailability and targeted delivery, controlled release kinetics tailored to pharmacological or nutraceutical applications, better protection against oxidation, light, and enzymatic degradation. The most commonly used encapsulating materials include: polysaccharides, gums, celluloses, lipids, proteins, and biopolymer blends (Muñoz et al. 2022). The selection of encapsulating agents depends on key physicochemical characteristics, such as: solubility, molecular weight, crystallinity, diffusion capacity, film-forming ability, emulsifying properties, viscosity, and Resistance to acidic or enzymatic conditions in the intestinal environment. Among the most applied encapsulation methods for cannabinoids are: coacervation, extrusion, molecular complexation, gelation, spray-drying, fluid bed coating, emulsification techniques, liposomes formation, and hot melt (Ortiz-Romero et al. 2021; Jiménez-Villeda et al. 2023). These techniques have been evaluated based on encapsulation efficiency, particle size, release behavior, and compatibility with food and pharmaceutical formulations. As such, micro- and nanoencapsulation stand out as critical tools for the industrial formulation of cannabis-based bioproducts, offering both functional and technological advantages in terms of controlled delivery and shelf-life extension.

## Bibliometric analysis

The main experimental developments useful in the conceptual recognition of this systematic review were presented in a sequential and systematic manner, all within the framework of worldwide recognition of the different techniques, methodological developments and research findings on the extraction, identification of metabolic pathways (metabolomics), concentration and micro/nanoencapsulation of metabolites from cannabis with chemical potential for use in the pharmaceutical and food industry. Through the use of office automation and information analysis tools, such as VOSViewer and tools of bibliographic databases, an analysis was made on the interaction between common terms, authors of greater relevance on particular topics and the worldwide distribution of publications derived from scientific research work.

**Fig. 4:**
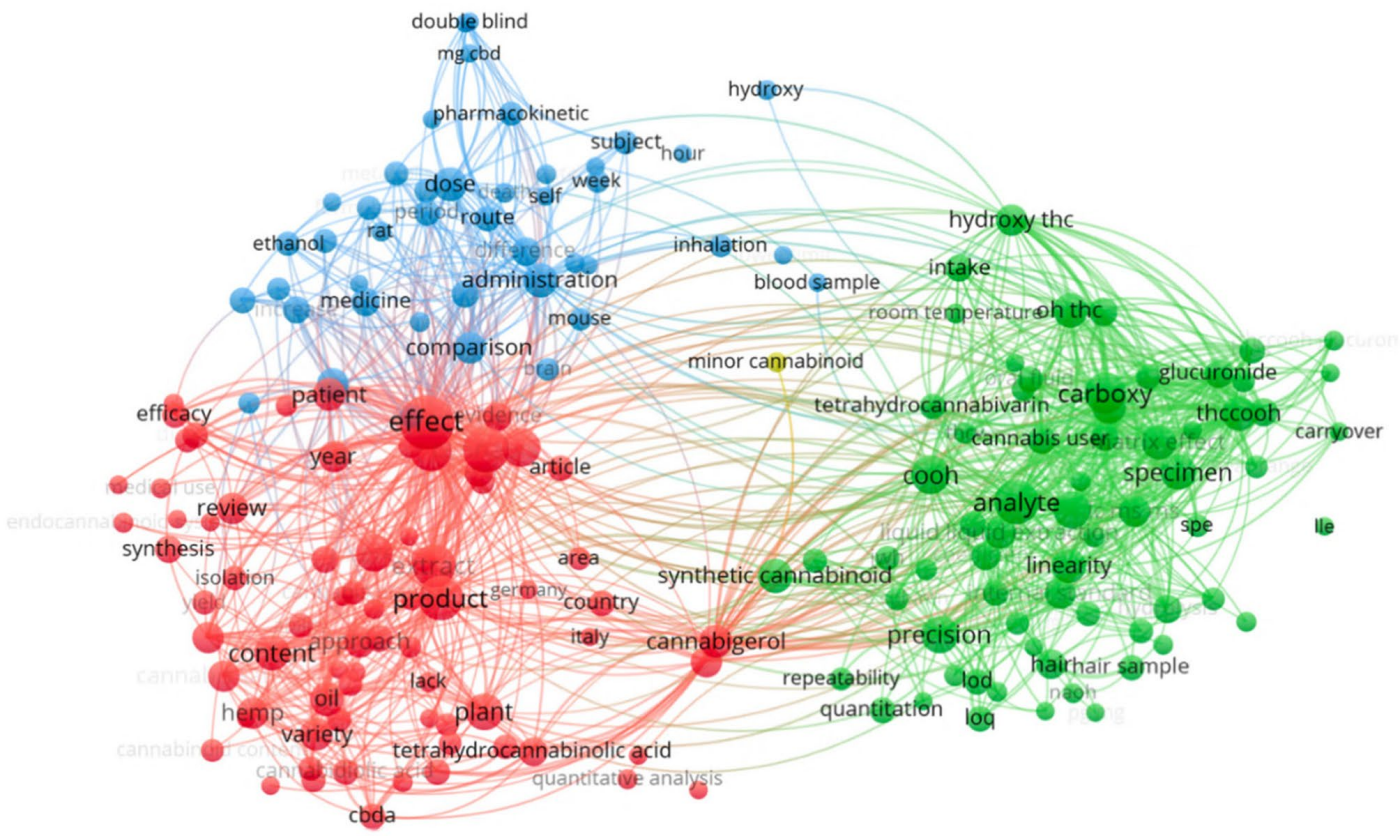
Scientometric analysis: Interaction between the most relevant and most repetitive terms in titles and abstracts of scientific research papers

Figure 4 represents a network of co-occurrence of terms, probably obtained from a bibliometric analysis of articles related to *Cannabis sativa*. The network shows three distinct thematic clusters, represented by nodes of different colors and connected by links indicating the relationship between terms. The red cluster (left), focuses on aspects related to the effects of cannabis and its products. Terms such as effect, patient, review, content and tetrahydrocannabinolic acid (THCA) are observed. This suggests that this group is related to studies on the effects of cannabis on patients, its content of active compounds and its use in medical and pharmaceutical contexts.

The blue group (upper left) is associated with pharmacokinetic and clinical studies. It contains terms such as double blind, pharmacokinetic, administration, dose, comparison and subject. This indicates that this group is linked to clinical trials, dose administration studies and the evaluation of the effects of cannabis in humans. The green group (right), on the other hand, focuses on chemical analysis and quantification methods of cannabinoids. It includes terms such as specimen, analyze, precision, hydroxy-THC, quantification and minor cannabinoid. This suggests that this group addresses studies on analytical techniques, metabolite identification and determination of compound concentrations in biological samples.

**Fig. 5:**
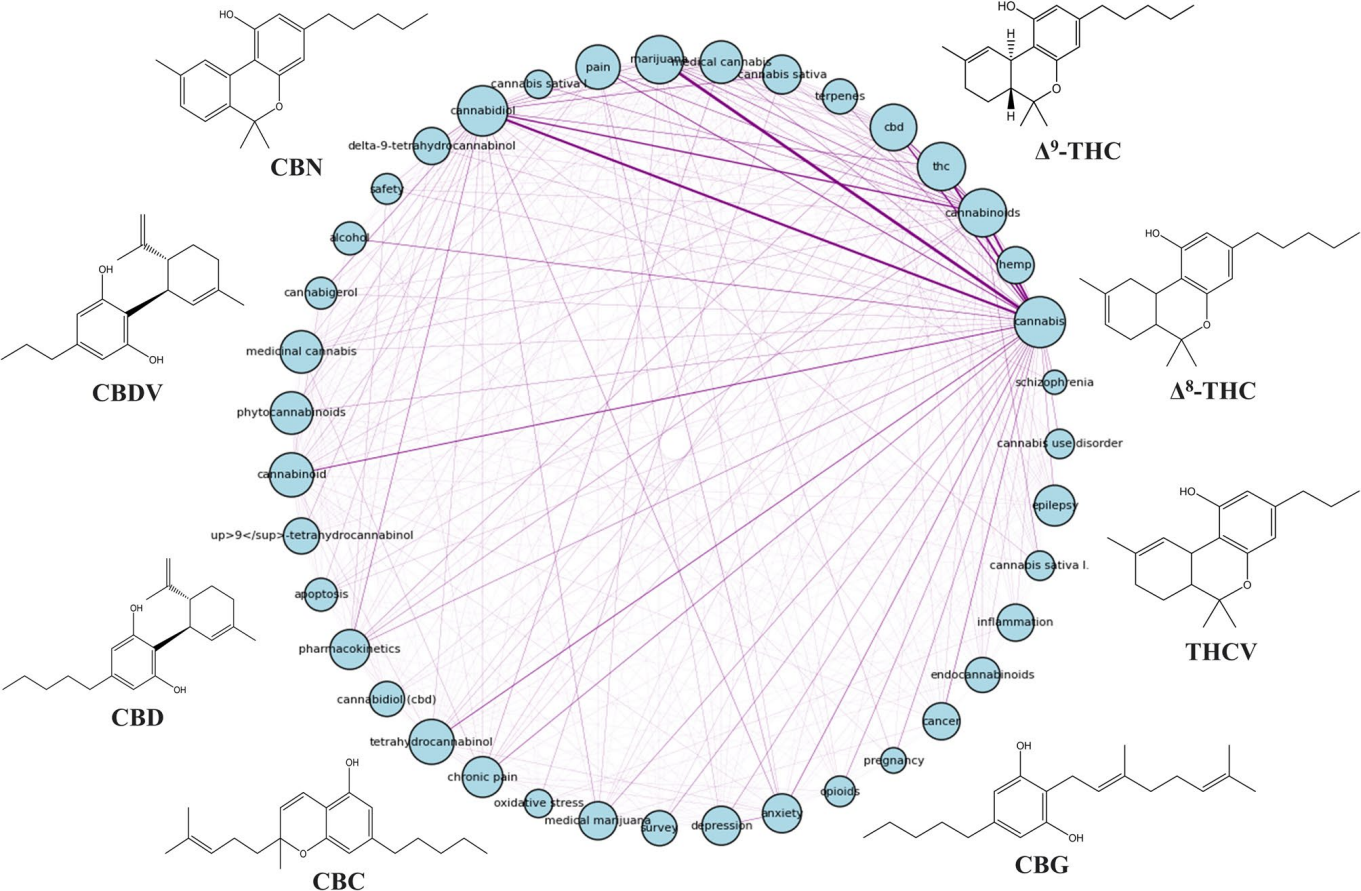
Circular keyword co-occurrence graph

Additionally, a network representation of relationships between cannabinoids and associated terms, probably obtained by bibliometric analysis, can be seen in Fig. 5. A circular structure is observed in which the blue nodes represent key terms, while the purple connections indicate the co-occurrence of these terms in the scientific literature. In the graph, three main areas are identified: effects of cannabis on patients, pharmacokinetic studies and chemical characterization. This graph focuses on the interaction between different cannabinoids (CBD, THC, THCV, CBC, CBN, CBDV, CBG) and their association with key terms in research. The Δ^9^-THC and CBD appear as central nodes with multiple connections, indicating their prominence in the scientific literature. Other cannabinoids such as CBG, CBC and CBN, which are emerging in recent studies for their potential therapeutic effects, are highlighted. The purple lines of different intensity suggest the strength of the connections between terms, reflecting the number of times they appear together in the literature.

**Fig. 6:**
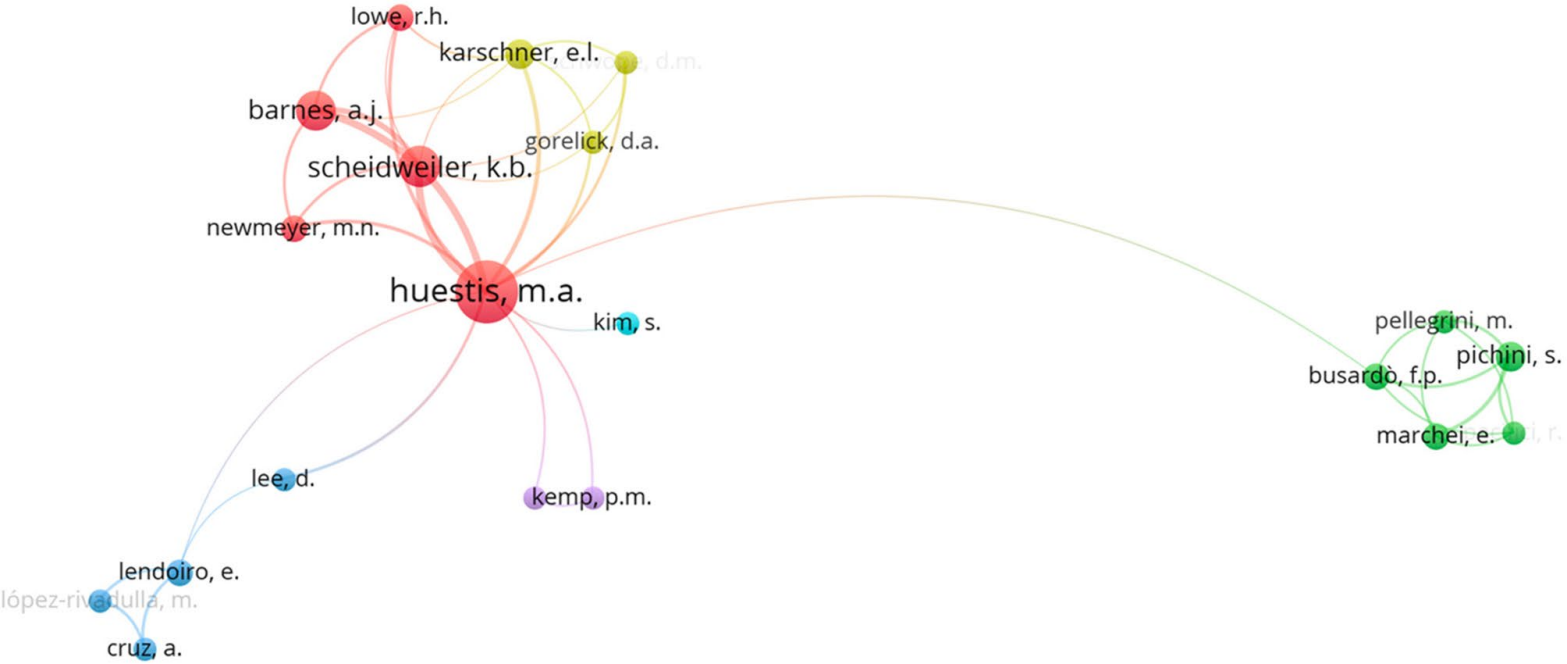
Analysis of the top exponents of scientific research production

Similarly, Fig. 6 shows a network of co-authorship in scientific research, where each node corresponds to a researcher and the connections indicate collaborations in publications. The colors differentiate collaboration groups, probably based on thematic areas or institutional affinity. The central node “Huestis, M.A.” is the most influential researcher in this network, with multiple connections to other authors, suggesting that he is a reference in the area of study (Huestis and Smith 2018). Around Huestis, M.A., there are several close collaborators such as Scheidweiler, K.B., Barnes, A.J., and Newmeyer, M.N., suggesting a consolidated research team (Marcotte et al. 2022). The blue group is more isolated and contains authors such as Lendoiro, E., and Cruz, A., indicating a possible different line of research or less frequent collaborations. The green group (led by Pellegrini, M. and Piccini, S.) is separate from the core group, but has a tenuous connection with Huestis, M.A., suggesting an indirect relationship between the two teams.

**Fig. 7:**
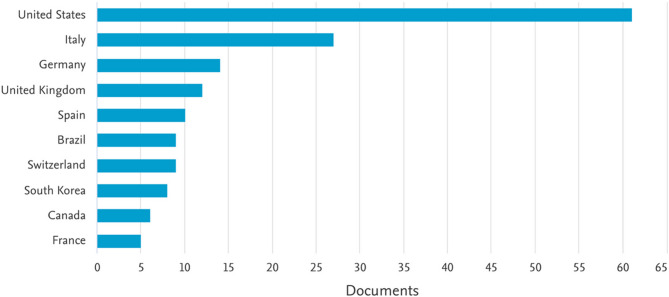
Worldwide distribution of scientific production

Figure 7 shows a bar chart showing the distribution of scientific papers by country, which allows us to identify which countries have contributed most to academic production in a specific area. The United States is, by a wide margin, the country with the most published papers in the subject analyzed, with approximately 60 publications. This indicates that most of the research in this field comes from U.S. institutions, probably due to their scientific infrastructure, funding and regulation of the subject. Italy is in second place with about 30 papers, followed by Germany with about 15. This suggests that Europe also has a significant participation in research on the topic, with relevant contributions from Germany, the United Kingdom, Spain and Switzerland.

For Latin America, Brazil is the only Latin American country present in the ranking, highlighting its emerging role in scientific research in this field. In Asia, South Korea is positioned with a lower production, indicating a more limited participation compared to the West. Therefore, in context, this graph shows which countries have led scientific production in this area. The predominance of the United States suggests that many of the methodologies, technologies and regulations reported come from that country. The presence of Italy and Germany may indicate that in Europe there is a strong focus on analytical, pharmacological or clinical studies. The results of the scientometric analysis show a relationship between the different terms with each field of study addressed in the agro-industrial chain of cannabis processing in the world, this said in the interaction between the different fields of work in the same: extraction, metabolomic analysis and micro/nanoencapsulation of active components and antioxidants from cannabis plants. The strong interaction in the development of works related to the effect on different impacts in the use of cannabis products: health, recreational, food and cosmetics is highlighted.

Additionally, it is important to highlight the relationship between the study of effectiveness, cannabinoids and analysis of their phytotherapeutic power; however, the formulation of bioproducts to meet the needs in pharmacological industrial chains, however, the use of cannabinoids for formulation are not directly related and are still little studied. On the other hand, it is evident that the scientific production related to the topics of the search chains specified in the development of the methodology is mostly concentrated in the United States, as is its maximum exponent in authorship, Huestis M.A.

## Knowledge gaps and future trends for research

Despite growing scientific and technological development in the cannabis processing industry, current research trends remain fragmented, with most studies focusing on isolated stages of the cannabis extract production pipeline—namely, extraction, metabolomics, and micro/nanoencapsulation. There is a notable lack of integration among these stages, particularly concerning terpenophenolic compounds with therapeutic potential for the formulation of pharmaceuticals and nutraceuticals. This disconnect limits the development of standardized, scalable bioproducts and hinders the translation of laboratory findings into real-world applications. While analytical techniques for profiling cannabinoids are advancing rapidly, research on biosynthetic pathways, especially for secondary metabolites with therapeutic relevance, remains limited. This represents a promising field for biotechnological innovation, particularly in the optimization of biochemical synthesis routes mediated by enzymes for cannabinoids such as cannabidiol (CBD), cannabigerol (CBG), and cannabinol (CBN).

Moreover, the formulation of functional food products and therapeutic agents represents a valuable strategy to harness the phytotherapeutic potential of cannabis-derived secondary metabolites. In this context, in vitro simulations of the human digestive system are essential to assess the bioaccessibility and bioavailability of cannabinoids under physiological conditions. Such studies could inform the development of micro/nanoencapsulated bioproducts capable of controlled and sustained release, offering personalized dosing and improved patient adherence—particularly for individuals with chronic or neurodegenerative conditions. One of the major technological challenges lies in extending the shelf life of cannabinoid extracts and ensuring their stability throughout storage and distribution. The application of encapsulation techniques using biodegradable and biocompatible materials, such as gelatin, chitosan, or polysaccharide blends, presents a viable solution. These materials offer protection from oxidative degradation and enable site-specific release in the gastrointestinal tract. Furthermore, low-temperature encapsulation technologies should be prioritized to preserve the structural integrity of thermolabile compounds like THC and CBD during processing.

Looking ahead, a comprehensive approach that combines extraction technologies, cannabinoid metabolomic profiling (cannabinomics), and advanced nanoencapsulation techniques will be crucial for the strategic development of cannabis-derived products for therapeutic and nutraceutical use. Future research priorities should include: establishing harmonized protocols that connect analytical chemistry with formulation science; exploring biosynthetic pathways to enhance the natural production of cannabinoids using enzyme-based strategies; evaluating innovative delivery systems such as nanoemulsions and stimuli-responsive carriers to improve targeted delivery and controlled release; and addressing safety, regulatory, and scalability considerations for encapsulated compounds (Al Ubeed et al. 2022). Ultimately, the progress of cannabis research depends on the integration of multiple disciplines—including biotechnology, pharmacology, food technology, and nanoscience—to support the responsible and science-driven use of *Cannabis sativa* in healthcare and nutrition.

## Conclusions

The thematic analysis developed in this systematic review evidences a growing interest in the extraction, metabolomic analysis, concentration and micro/nanoencapsulation of metabolites with phytotherapeutic potential. In recent years, these areas have gained relevance in the scientific community due to their impact on the production of knowledge about *Cannabis sativa* and its applications in health. The predominant focus in the literature is on the effects of cannabis in the population, addressing both its recreational and therapeutic use, especially through the characterization and formulation of specific extracts. However, the integration of methodologies and protocols to increase added value in the agro-industrial cannabis processing chain remains a challenge little addressed in the scientific literature. Although extraction techniques have been studied at the laboratory scale to optimize the production of bioproducts and extracts with pharmaceutical and nutraceutical potential, there is still a gap in the application of this knowledge at the industrial level. The lack of studies that directly correlate extraction operating conditions with the efficiency and standardization of final products hinders their large-scale implementation.

The analysis of the information gathered in this review allowed the identification of thematic relationships between the different lines of research on cannabis at the global level. However, a more in-depth evaluation of published patents would provide a more accurate perspective on the current state of the industry in terms of extract production. This would include analysis of yields, comparison of methodologies and protocols used, as well as identification of operational variables that influence process efficiency. In this sense, future research may benefit from a greater articulation between technological development and applied research, in order to establish production models that link extraction optimization with controlled dosing of specific metabolites. This approach would improve the traceability and safety of *Cannabis sativa* extracts, regardless of the route of administration, thus contributing to the advancement of the agro-industrial and pharmaceutical sector.

## Data Availability

Not applicable.
